# Influence of different caregiving styles on fundamental movement skills among children

**DOI:** 10.3389/fpubh.2023.1232551

**Published:** 2023-11-28

**Authors:** Jiahui Hu, Shudan Zhang, Weibing Ye, Yuanye Zhu, Huiling Zhou, Lihua Lu, Qian Chen, Mallikarjuna Korivi

**Affiliations:** ^1^Institute of Human Movement and Sports Engineering, College of Physical Education and Health Sciences, Zhejiang Normal University, Jinhua, China; ^2^Handu Xinyuan, No. 1 Primary School, Xi'an, China; ^3^The Affiliated Kindergarten of Jinhua Polytechnic, Jinhua, China; ^4^Zhejiang Sports Science Institute, Hangzhou, China

**Keywords:** preschoolers, motor skills, parenting, grandparenting, ball skills, physical activity

## Abstract

**Purpose:**

This study investigated the influence of parenting and grandparenting caregiving styles on fundamental motor skills (FMS) of preschool children.

**Method:**

A total of 1,326 preschool children (698 boys, 628 girls) aged 4–6 years were recruited from the kindergartens of Jinhua City, China. Locomotor skills (LM), ball skills (BS), and total fundamental movement skills (TS) of children were assessed by the Test of Gross Motor Development-3rd edition (TGMD-3).

**Results:**

There were 978 children in parenting and 348 children in grandparenting caregiving styles. The LM, BS and TS scores of children were considerably (*p* < 0.001) increased with age (irrespective of sex or caregiving style). For the sex comparisons, BS scores of boys were significantly higher than girls (*p* < 0.001), while LM and TS scores were not different between boys and girls. For the caregiving style comparison, parenting is superior to grandparenting in developing of children's FMS. Parenting boys of 4-, 5-, and 6-years old showed better BS compared to age-matched parenting girls, whereas boys of 5-years old in grandparenting only showed better BS compared to same-age grandparenting girls (*p* < 0.05). Furthermore, parenting boys of 6-years reported higher LM (*p* < 0.01), BS (*p* < 0.001), and TS (*p* < 0.001) scores compared to grandparenting boys, but girls' FMS at all ages were not significantly different between the caregiving styles.

**Conclusion:**

Parenting caregiving style is positively associated with proper development of FMS among children. Girl children with poor FMS in grandparenting may need a special care or intervention programs to promote their FMS.

## 1 Introduction

Fundamental movement or motor skills (FMS) are defined as basic learning movement patterns in preschool children. Preschool age is a crucial period for developing and practicing of FMS that are essential for more complex physical and sporting activities (1). The items in FMS, including locomotor skills, object control, and stability skills, are positively correlated with many aspects of children's health (2). Children with poor FMS are often fail to attain specific motor skills, which induce physical inactivity that lead to anxiety and depression (3–5). It is further showed that elementary school children with poor FMS had learning disabilities and cognitive impairments (6, 7). On the other hand, children with higher levels sof FMS competence reported to have better cognitive function and academic performance (7, 8). This scenario emphasizes that preschool age or early childhood is a critical period for FMS learning as well as teaching (1). FMS strongly mediates specific motor skills of children at an early age, and its impact may persist until reaching adolescence or even a lifetime (9). During early childhood, several domains, such as biological factors (sex, age, and bodyweight), socio-economic status, cultural background and caregiving styles, are involved in proper development of FMS (10, 11). Among these domains, caregiving style or parenting is one of the important factors that could influence FMS development, and this phenomenon is yet to be disclosed in preschool children.

Previous studies have shown that family environment, particularly parenting, or caregiving style is involved in improving of physical activity, academic performance and motor skills among children (8, 11, 12). Since FMS cannot occurs naturally (13), caregivers' role is inevitable for proper learning of motor skills in the early stage of childhood. Very recently, high parental participation has been shown to improve FMS proficiency in children of 2–7 years old (14). In a family system, parenting and/or grandparenting is common in several countries, and grandparenting has been increased worldwide in the recent decades (15). About 80% of adults aged 65 years and older are grandparents in the USA, where grandparenting is becoming more intense (16). Most of grandparents are involved in almost every aspect of the daily care and support of their grandchildren in the USA (17). Reliance on grandparents for caring of children is also prevalent in Australia (18), Canada (19), England (20), and many other countries (21). In the Confucian culture, taking care of grandchildren is more common among Chinese grandparents (22). In addition, young parents in China tend to move to cities due to modernization or employment, which results in leaving of their preschool children with grandparents (23). Data from the China Health and Retirement Longitudinal Study (CHARLS) revealed that about 53% of grandparents in China are providing care for their grandchildren, aged under 16 years (24).

According to the social-ecological model and theoretical frameworks, parenting (by parents or grandparents) plays a fundamental role in shaping their children's behavior and personality (25, 26). Parenting is broadly conceptualized into two dimensions, including autonomy-supportive and controlling styles (25, 27). Autonomy-supportive parenting is characterized by empathy for children and respect for their perspectives, while controlling parenting (psychological or behavioral controlling) ignores child's opinions, needs and feelings (28, 29). Autonomy-supportive parenting is associated with positive outcomes (less depression, higher wellbeing, and self-efficacy), whereas controlling parenting is associated with negative outcomes (social incompetence, anxiety, and depression) among children (25, 28–30). Autonomy-supportive parenting was said to be associated with improved child wellbeing and family cohesion in German families during COVID-19 pandemic (31). Not only children, but also toddlers benefited from the autonomy-supportive parenting, as toddlers' rule internalization was positively correlated with authoritative parenting practice (32). Furthermore, parents' autonomous motivation with positive emotions was directly associated with students' positive emotions and self-efficacy for doing homework (33). Based on the above two dimensions, parenting is further classified into four types, namely authoritarian, authoritative, permissive, and uninvolved types (27, 34, 35). Among these, authoritative parenting type is a balanced one, in which parent develops a close, nurturing relationship with their children, and children are confident, responsible, and able to self-regulate as they grown (35). Irrespective of parenting type, caregivers, either parents or grandparents play a vital role in supporting and promoting the motor development in children from an early age (14, 36). Previous studies identified the differences in FMS proficiency between boys and girls, which might be due to their behavioral habits, biological or social factors (37, 38). A meta-analysis showed that age of children (3–6 years) is correlated with gender specific differences in object control skills (38), but parenting role is unanswered in their analysis. Therefore, it is important to assess the FMS proficiency in boys and girls separately from their early childhood, in both parenting and grandparenting caregiving styles. Identifying of a child, who do not masterly perform his/her FMS, is important for designing of appropriate interventions based on their age, sex, and caregiving style.

Although parenting and grandparenting is advantageous on various outcomes of children, the child caring values and benefits of caregiving styles are contradictory between parents and grandparents (39). The outcomes (social, educational, physical, and health) of preschool children are reported to be equivocal with parenting and grandparenting. For example, a study showed that parental involvement can decrease behavioral problems of children and improve their social skills (40). Luby et al. (41) stated that maternal support in early childhood is a predictive of larger hippocampal volumes among children at school age. In contrast, some studies showed negative effects of grandparenting on children's creativity (42) and bodyweight (overweight) (43), and beneficial effects on infants' (9 months) communication and personal-social development (44). Nonetheless, the influence of grandparenting on children's FMS is remaining unexplained. As grandparenting is continuously increasing in China (23, 24), there is a need of studying children's motor skill development, and it is important to unveil whether grandparenting caregiving style could affect preschool children's FMS.

In this study, we aimed to monitor the FMS development in kindergarten children (aged 4–6 year), and compare the influence of parenting and grandparenting caregiving styles on FMS competence. We then examined the age-specific and sex-specific changes in FMS competence among children with both caregiving styles. Since authoritative parenting is favor to promote several outcomes in children (40, 41), we hypothesized that authoritative parenting might be beneficial to develop FMS proficiency, and this development might be associated with age or sex of children.

## 2 Materials and methods

### 2.1 Recruitment strategy and participants

This cross-sectional study was conducted in Jinhua, a city with ~1 M population in Zhejiang Province of Eastern China. A total of 1,343 healthy children (4, 5, and 6 years old) from two large-scale kindergartens (affiliated to Zhejiang Normal University and Jinhua Polytechnic) were recruited by a random cluster sampling method. The study was conducted from March to June 2021. During the study period, 17 children were dropped due to incomplete assessment scores, resulting 1,326 children as the final sample size. The basic characteristics of children, including age, height, weight, body mass index (BMI) and sex were recorded at their respective kindergartens. There were a total of 698 boys and 628 girls. The caregiving style of each child either parenting or grandparenting was recorded with the help of their teacher or caregiver. Other variables, like family economy status and sex of caregivers were not analyzed. Children in a family with a combination of caregivers (parents and grandparents) were not enrolled, and the ideal parenting type in this study was “authoritative.”

The details of this study were provided with the Chinese version of the written informed consent form and also explained verbally to the directors of the kindergarten, teachers, parents, or grandparents, and students before their voluntary participation. The study design and all assessment procedures were reviewed and approved by the Institutional Ethical Committee of Zhejiang Normal University, and the approval number is ZSDR2019013.

### 2.2 Assessment of basic characteristics of children

The basic characteristics, including bodyweight (kg) and height (m) of children were measured using a weight and height scale to the nearest 0.1 kg and 0.1 cm, respectively. Then BMI was calculated using the weight and height of children at respective ages [weight (kg)/height (m)^2^]. These assessments were performed in the empty hall of each kindergarten during the school hours. Meanwhile, demographic data (age and gender) of children provided by their teachers were recorded for the analysis. Caregivers provided the caregiving details of their children. Based on the caregiving type, we categorized the children into two groups namely, grandparenting and parenting. In “grandparenting caregiving style” grandparents are the main caregivers, while in “parenting caregiving style” parents are the main caregivers. The main caregiver means the person, who looks after the child's daily activities at least from 6 months prior to the study. Taking care of children by relatives, guardians or babysitters were excluded from our study.

### 2.3 Assessment of motor skills of children

In this study, FMS performance of all age groups of children was assessed using the scale, Test of Gross Motor Development, 3^rd^ edition [TGMD-3, (45)]. The TGMD-3 includes two skill categories: six locomotor skills (i.e., running, galloping, sliding, skipping, horizontal jumping, and hopping) and seven ball skills (i.e., overhand throwing, underhand throwing, two-hand catching, one-hand stationary dribbling, forehand striking of the self-bounced ball, two hands striking a stationary ball, and kicking a stationary ball). For the test evaluation, each skill was categorized into 3–5 components, and each component was scored as either 1 (present) or 0 (absent). There were 46 points for locomotor skills (LM) and 54 points for ball skills (BS), with a full score of 100. The children's FMS competence test was conducted in the indoor gymnasium of each kindergarten after obtaining permission from the administrators. Two motor skill subset scores (LM and BS) were computed from the sum of raw scores from each subset, and the total fundamental movement skills score (TS) was the sum of LM and BS.

The TGMD-3 scale used in this study is a valid and reliable assessment tool for measuring the Chinese kindergarten children's FMS performances, and reported to have good test-retest reliability and internal consistency (46, 47). A total of 14 professionals, including two sport science researchers, two psychologists and 10 testers (postgraduates) were involved in the test assessments. The administration of TGMD-3 for the assessment required roughly ~20 min for each child. Two independent testers simultaneously observed each child's performance. The correlation coefficient between the testers was used to ensure the consistency for different rater scores when scoring the same subject and the results proved that the inter-rater reliability was good. All assessments were conducted in accordance with the TGMD-3 recommendations (48, 49). Required equipment was prepared prior to the assessment date. On the day of assessment, all procedures were verbally explained, and accurate demonstration was given on scoring of each skill. Each child completed one practice, and then performed two formal trials. The scores of two formal trials were recorded and used for the analyses.

### 2.4 Statistical analysis

Obtained data were analyzed using the IBM SPSS Statistics (Version 22.0, IBM Corporation, New York, USA). Descriptive statistics for participant characteristics were displayed as Means ± SD or *n* (%). For the purpose of analysis, children were grouped on the basis of sex (two groups), caregiving styles (two groups), and age (three groups). For the age comparisons, all children were arbitrarily categorized into three age groups, including 4, 5, and 6 years. One-way ANOVA was performed (separately) among the FMS scores and caregiving styles, age, and sex to explore the influence of each of these three variables. In order to examine any significant difference across caregiving styles according to age groups and sex classes, across age groups according to caregiving styles and sex, and across sex classes according to caregiving styles and age groups, one-way ANOVA was subsequently performed. The results of the one-way ANOVA displayed through the *F*-values and level of significance was set at *p* < 0.05.

## 3 Results

### 3.1 Characteristics of children

In this study, a total of 1,326 kindergarten children aged 4, 5, and 6 years with parenting and grandparenting caregiving styles were participated. There were 522 boys and 456 girls in parenting, and 176 boys and 172 girls in grandparenting caregiving style. The average height of boys and girls in parenting and grandparenting caregiving styles was not different (Table 1). Similarly, the weight and BMI of children was not differing either with parenting or grandparenting at respective ages. We noticed an increased height and weight of children by age in both caregiving styles; however, we did not notice such a trend in BMI (Table 1).

**Table 1:** Basic characteristics of the preschool children.

	**Parenting**	**Grandparenting**	**Total**
Number of children	978 (73.8%)	348 (26.2%)	1,326 (100%)
Boys	522 (74.8%)	176 (25.2%)	698 (100%)
Girls	456 (72.6%)	172 (27.4%)	628 (100%)
Age (y)			
4	115 (69.3%)	51 (30.7%)	166 (100%)
5	330 (68.3%)	153 (31.7%)	483 (100%)
6	533 (78.7%)	144 (21.3%)	677 (100%)
**Height (m)**			
Boys	1.18 ± 0.08	1.17 ± 0.08	1.18 ± 0.08
Girls	1.19 ± 0.07	1.18 ± 0.07	1.19 ± 0.07
Age (y)			
4	1.06 ± 0.07	1.08 ± 0.05	1.07 ± 0.07
5	1.16 ± 0.05	1.17 ± 0.05	1.16 ± 0.05
6	1.22 ± 0.08	1.23 ± 0.05	1.23 ± 0.05
**Weight (kg)**			
Boys	20.64 ± 3.80	20.17 ± 3.19	20.52 ± 3.66
Girls	20.57 ± 3.58	20.46 ± 3.31	20.54 ± 3.51
Age (y)			
4	17.16 ± 2.31	17.54 ± 1.62	17.27 ± 2.12
5	19.44 ± 2.03	19.53 ± 1.87	19.47 ± 1.98
6	22.07 ± 3.98	22.13 ± 3.77	22.08 ± 3.94
**BMI (kg/m** ^2^ **)**			
Boys	14.70 ± 1.46	14.57 ± 1.46	14.66 ± 1.46
Girls	14.54 ± 1.62	14.53 ± 1.45	14.54 ± 1.57
Age (y)			
4	15.24 ± 1.18	15.13 ± 1.02	15.21 ± 1.13
5	14.41 ± 1.28	14.28 ± 1.29	14.37 ± 1.28
6	14.62 ± 1.54	14.62 ± 1.67	14.62 ± 1.70

y, years; m, meter; kg, kilogram; BMI, body mass index; kg/m^2^, kilogram/meter^2^. Values expressed in numbers, percentage and mean ± standard deviation.

### 3.2 Differences in FMS scores among children by age, sex, and caregiving styles

The scores of locomotor skills, ball skills, and total fundamental movement skills of children were presented in Table 2. We found that LM, BS, and TS scores of children were progressively increased with age (*p* < 0.001). For the gender comparison, the scores of LM and TS were not significantly different between boys and girls, whereas the BS scores were significantly higher in boys compared with girls (*p* < 0.001). For caregiving styles, children with parenting showed superior FMS scores (LM, BS, and TS) than that of children with grandparenting caregiving style (Table 2).

**Table 2:** Changes in FMS scores among children by age, gender, and caregiving styles.

**FMS**	**Age (y, M** ±**SD)**			**F (d.f. = 2)**	**Sex (M** ±**SD)**		**F (d.f. = 1)**	**Caregiving styles (M** ±**SD)**		**F (d.f. = 1)**
	**4**	**5**	**6**		**Boys**	**Girls**		**Parenting**	**Grandparenting**	
LM	20.65 ± 7.10	25.53 ± 7.96	31.04 ± 6.08	189.64^***^	27.58 ± 7.90	27.87 ± 7.84	0.44	28.10 ± 7.83	26.65 ± 7.90	8.68^**^
BS	15.52 ± 6.15	20.59 ± 6.66	28.16 ± 7.24	308.00^***^	24.60 ± 8.66	22.91 ± 7.90	13.69^***^	24.57 ± 8.55	21.66 ± 7.34	31.85^***^
TS	36.17 ± 11.55	46.12 ± 12.88	59.20 ± 11.36	328.11^***^	52.18 ± 14.93	50.78 ± 14.22	3.05	52.66 ± 14.73	48.31 ± 13.78	23.13^***^

LM, locomotor skills score; BS, ball skills score; TS, total fundamental movement skills score; d.f., degrees of freedom; The scores were significant at ^**^p < 0.01 and ^***^p < 0.001 in each factor (age, sex, and caregiving style).

### 3.3 Changes in FMS scores of boys and girls with different caregiving styles

**Figure 1:**
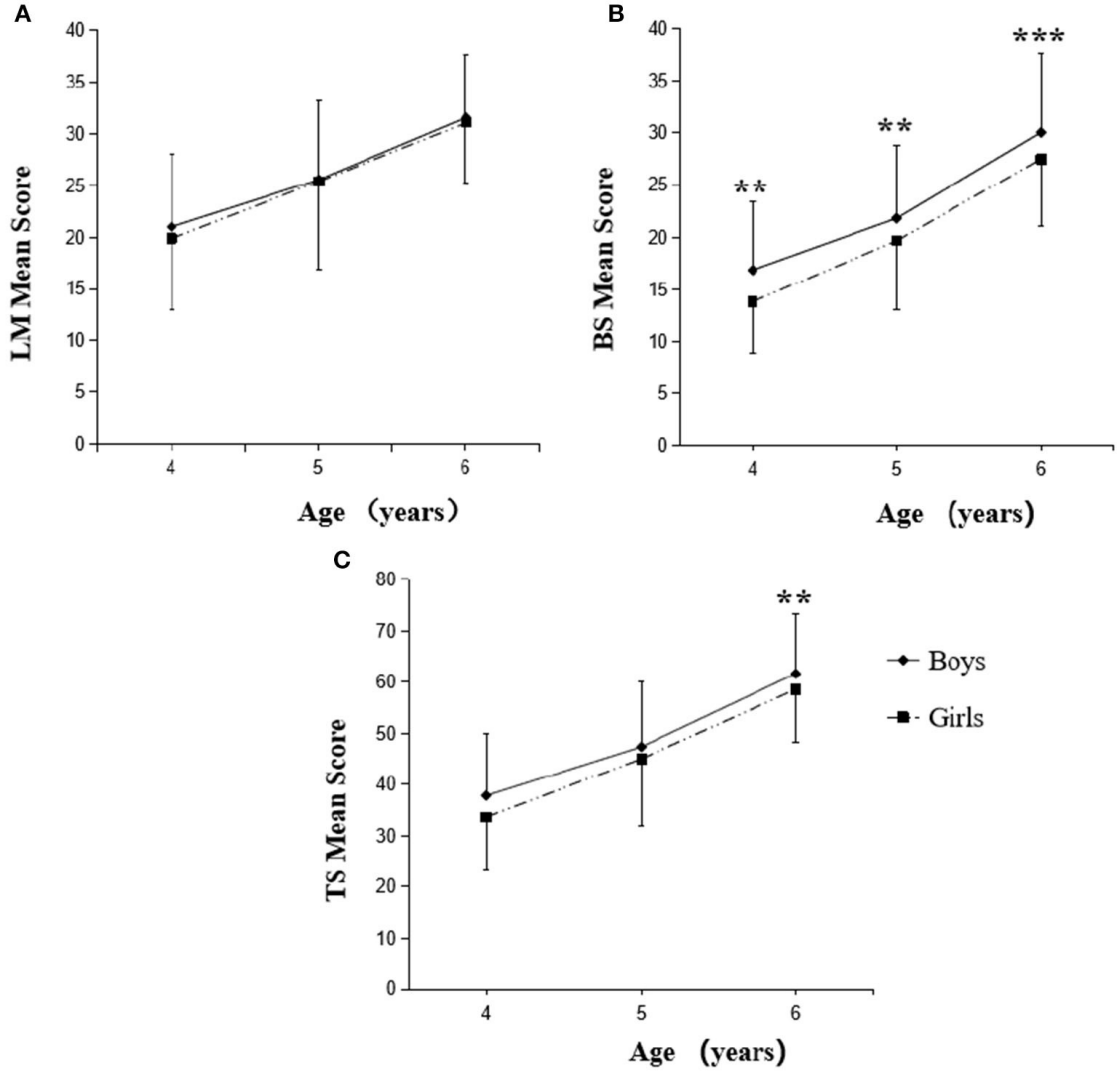
Changes in FMS scores of boys and girls with parenting caregiving style. LM, locomotor skills **(A)**; BS, ball skills **(B)**; TS, total fundamental movement skills **(C)**. The scores are significant (**P < 0.01, ***P < 0.001) between boys and girls at respective age.

**Figure 2:**
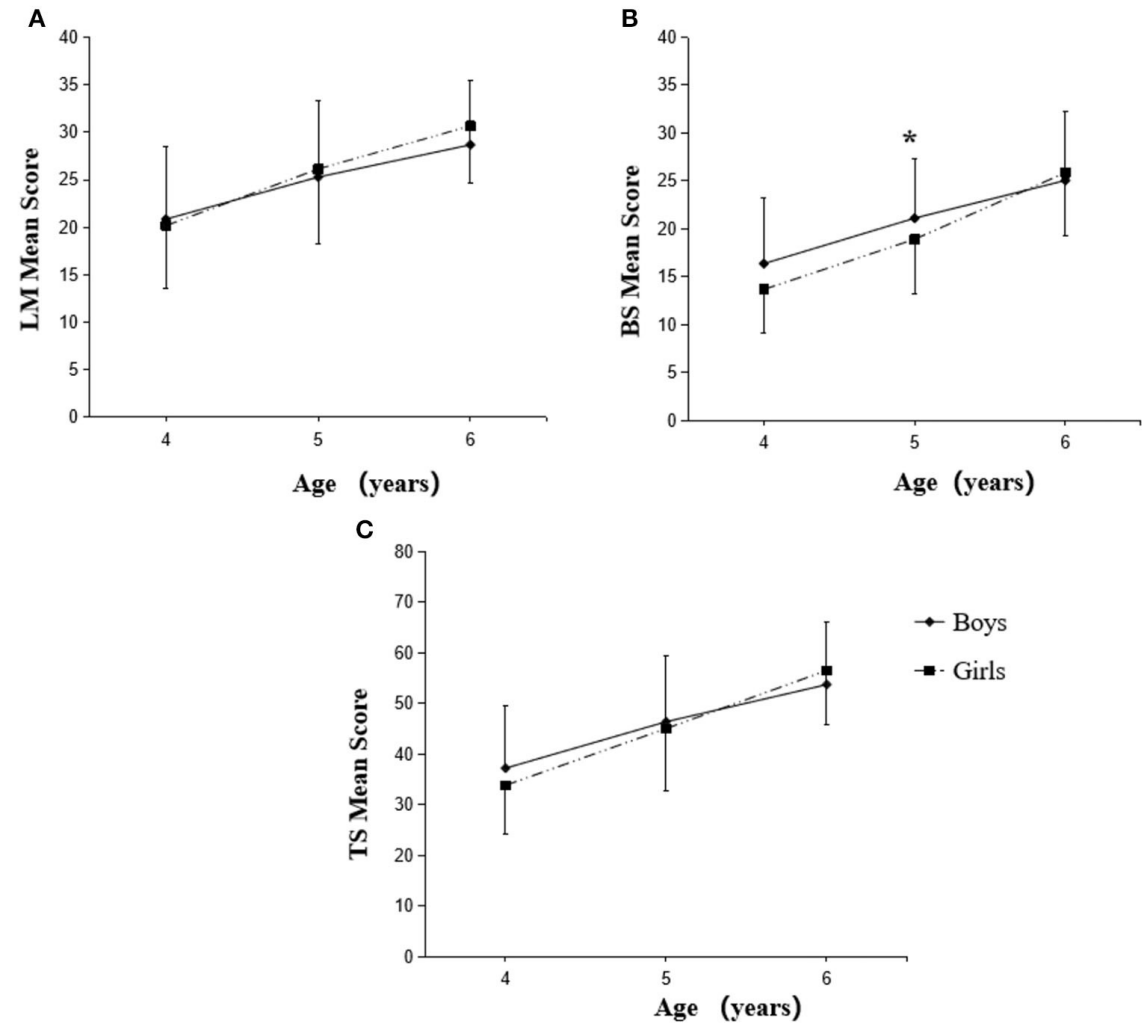
Changes in FMS scores of boys and girls with grandparenting caregiving style. LM, locomotor skills **(A)**; BS, ball skills **(B)**; TS, total fundamental movement skills **(C)**. The scores are significant (*P < 0.05) between boys and girls at respective age.

The FMS performance scores of boys and girls with parenting caregiving style are shown in Figure 1. We noticed that the progressive increase of LM scores with age was not significantly different between boys and girls in parenting (Figure 1A). However, the BS scores of boys at each age (4, 5, and 6 years) in parenting were significantly higher than that of girls at respective ages (Figure 1B). Nevertheless, boys at the age of 6 years only showed the higher total FMS scores (*p* < 0.01) than girls at the same age (Figure 1C). In grandparenting caregiving style, the progressive increase of LM and TS scores with age was not significantly different between boys and girls (Figures 2A, C). Five-year-old boys in grandparenting only showed the higher BS scores (*p* < 0.05) compared to girls at the same age, which is different from the boys in parenting (Figure 2B).

### 3.4 Parenting caregiving style promotes FMS in boys, not in girls

**Figure 3:**
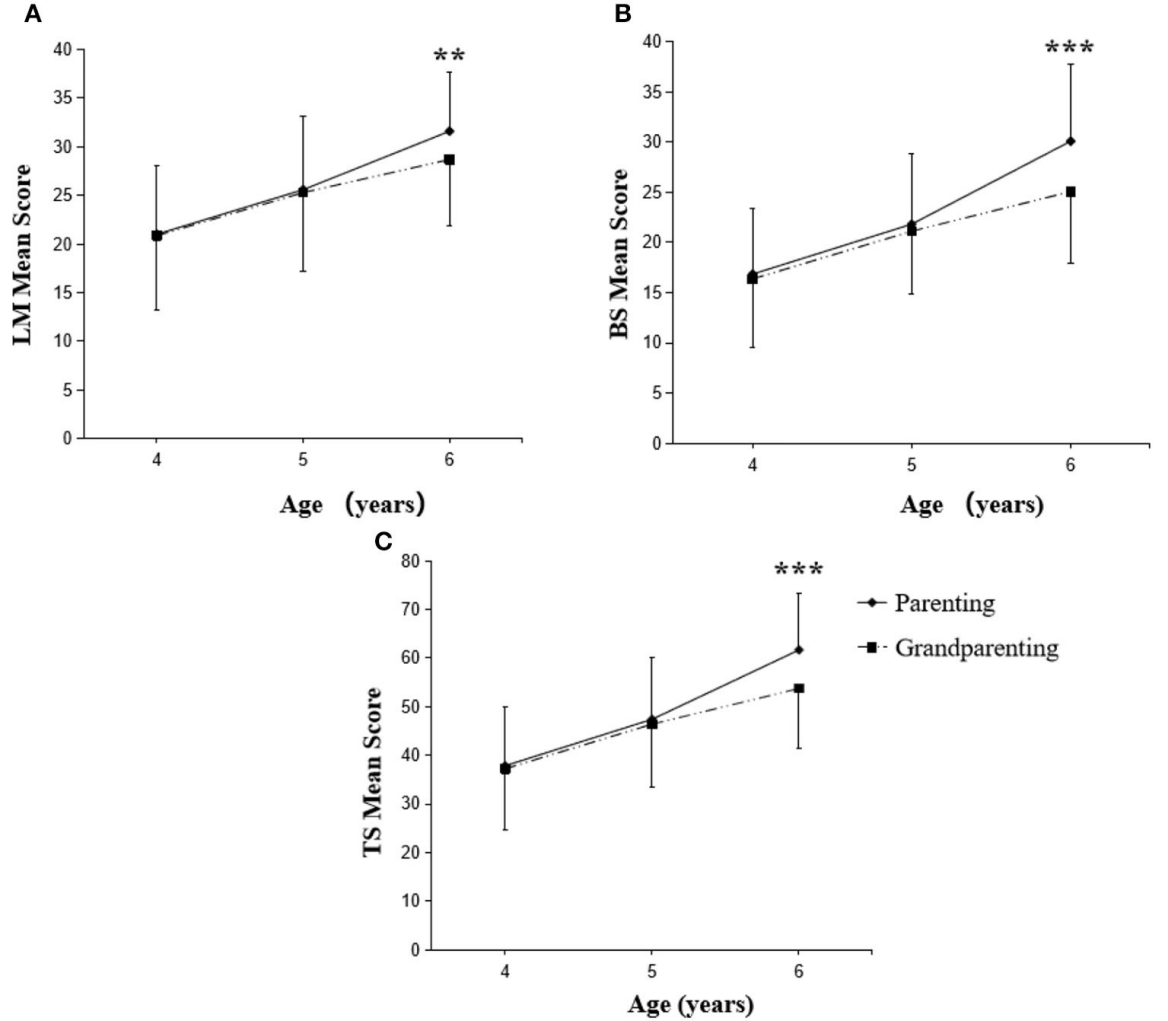
Changes in FMS scores of boys with parenting and grandparenting caregiving styles. LM, locomotor skills **(A)**; BS, ball skills **(B)**; TS, total fundamental movement skills **(C)**. The scores are significant (**P < 0.01, ***P < 0.001) between parenting and grandparenting children at respective age.

**Figure 4:**
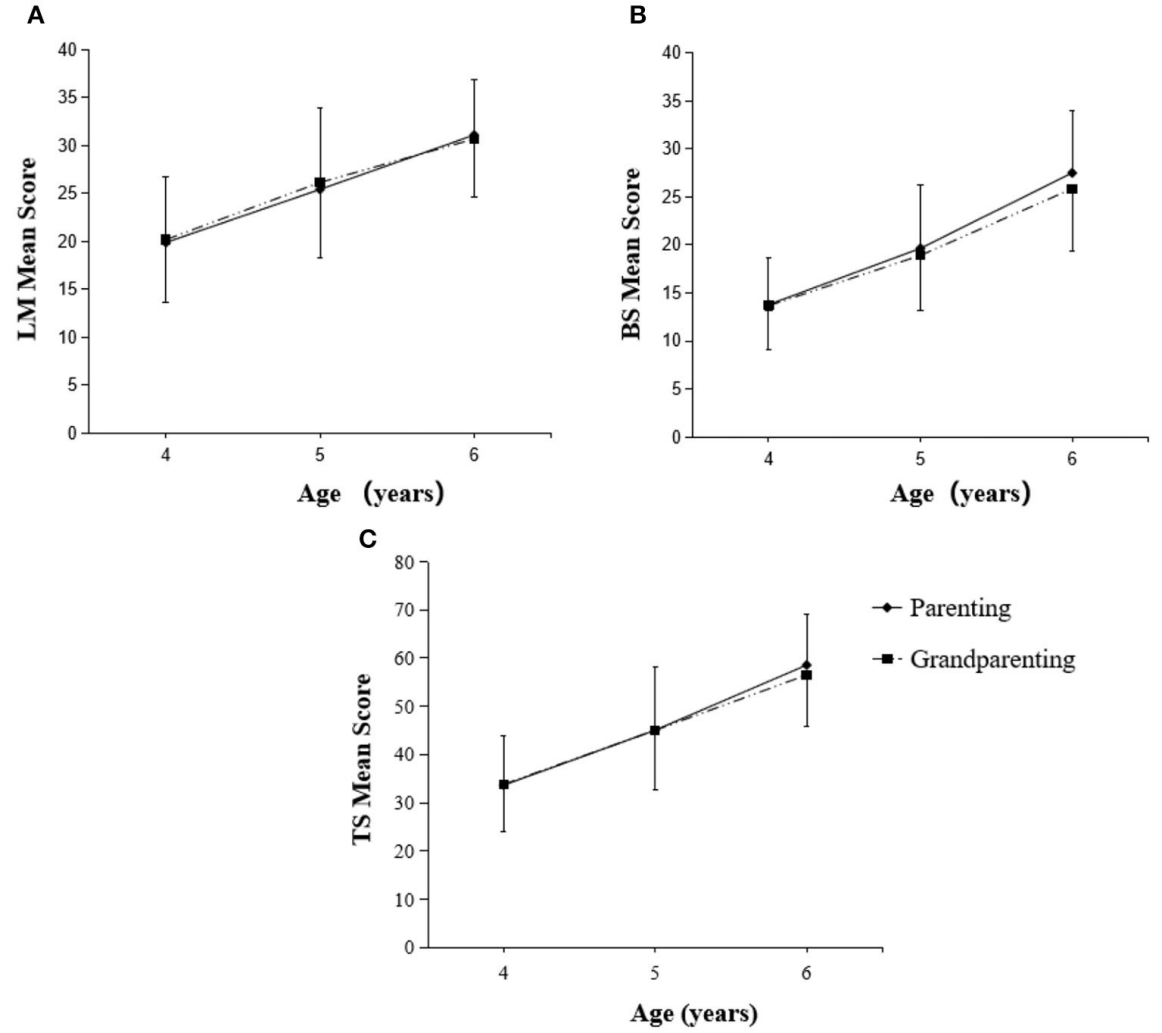
Changes in FMS scores of girls with parenting and grandparenting caregiving styles. LM, locomotor skills **(A)**; BS, ball skills **(B)**; TS, total fundamental movement skills **(C)**.

Figures 3, 4 showed the influence of caregiving styles on development of FMS among children of 4–6 years old. Initially, we found that there was no significant difference between two caregiving styles on LM, BS, and TS scores of boys at the age of 4- and 5-year. However, boys at the age of 6-year in authoritative parenting reported significantly higher LM (*p* < 0.01), BS (*p* < 0.001), and TS (*p* < 0.001) scores compared with that of same age of boys in grandparenting (Figure 3). It is worth to note that the FMS scores of girls (LM, BS, and TS) were not specifically influenced by either parenting or grandparenting caregiving style (Figure 4).

## 4 Discussion

To the best of our knowledge, this is the first study to explore whether caregiving style (parenting and grandparenting) could influence the fundamental motor skills of preschool age children. We studied the changes in FMS among children (4–6 years) from Zhejiang province of China, and the changes were compared with their age, sex, and caregiving styles. Our findings showed that caregiving styles influence preschool children's FMS, and this influence is associated with age (4–6 years) and sex of children. Parenting caregiving style significantly improved FMS of boys at 6-year, while FMS of girls (all ages) remained unchanged with parenting and grandparenting caregiving styles. To be specific, the ball skills of boys at all ages (4-, 5-, and 6-year) were better than girls of respective ages with parenting caregiving style. Our findings revealed that authoritative parenting type is important to promote various motor skills of preschool children. However, the influence of parental sex, and the role of other parenting types (authoritative, permissive, and uninvolved) on children's FMS competence is yet to be elucidated.

### 4.1 Age-specific response of FMS

Increasing age is the most consistent key variable of all subsets of FMS among children (50). In our study, the scores of FMS of children aged 4–6 years were significantly improved with age. Our results are consistent with previous findings of Bolger et al. (51), who reported increased locomotor and object-control skills in Irish children aged 5–10 years. The early stage of children (3–7 years) is a critical period for proper development of FMS (1), and improvement of FMS after this age may not be efficient as early stages (52). The possible explanation is that children have different physical development characteristics in different stages of growth. During this period, children have more opportunities to engage in several physical activities for daily needs and thereby learn more motor skills. Therefore, the motor proficiency of children constantly increases with age, which then leads to a gradual increase in both locomotor skills and ball skills naturally (51). From our findings, it is interesting to note that the improvement of ball skills of girls with age was comparatively lower than that of the improvement of ball skills of boys with age.

### 4.2 Sex-specific response of FMS

Exploring of sex-specific responses of FMS among children with parenting and grandparenting is an important perspective to understand whether caregiving style affects FMS differently in boys and girls. In this context, we found that boys scored higher than girls in BS but not in LM and TS. We then identified that boys of all ages (4-, 5-, and 6-year), particularly in parenting caregiving style, possess better ball skills than girls. Similar to our findings, a study from Australia reported that the boys had better object-control skills than the girls (53). Other studies from the USA (54) and Brazil (55) also reported superior scores of BS and LM in boys than that of in girls. In contrast, some studies reported specific motor skills of girls are better than boys. For instance, the jumping skills of Belgian girls are superior than boys (56), and the fundamental skill performance of girls from Hong Kong was better than boys (57). The difference in FMS subsets between boys and girls might be linked with various factors. Gender differences among children in learning of FMS depend on their family, elder siblings, peers, and teachers through socialization and imitation, and consequently participate in particular activities that fit these gender norms (38). A study on children from the deprived areas of England stated that cultural background, ethnicity, and other factors are critically involved in gender-specific differences in learning of motor skills (58). The better ball skills of boys in our study explained that boys may spend more time than girls in practicing of object-control related activities, like ball games, and thereby develop their ball skills. In addition to the above said factors, we found caregiving style is also a considerable factor for the sex-specific differences in various motor skills among preschool children.

### 4.3 FMS and caregiving styles

According to Clark (59), it is a common misconception that children's FMS develop naturally as they grow-up. It is also said that providing of well-equipped physical activity facility may not improve FMS in children, instead being engaged, accompanying person, or supporting sibling may contribute for appropriate development of FMS (1, 60). Goodway et al. (1) also pointed out that FMS proficiency does not occur naturally with growth and development of children, but it requires structured teaching, practice, and consolidation. Although social-environmental variables influence the FMS, parental and family-related variables (61) or older siblings (62) of children are strongly involved in proper development of FMS. For the first time, we demonstrated the influential role of caregiving style among Chinese children in developing of motor skills. Our findings showed that boys in parenting at the age of 6-year performed better LM, BS, and TS scores than the same age of boys in grandparenting. Interestingly, caregiving style had no effect on the development of FMS proficiency among girls.

There are several reasons to explain the differences in children's FMS scores between parenting and grandparenting caregiving styles. The selected parenting type in this study was authoritative, in which parents maintain a positive and warmth relationship with their child, and child tend to gain confidence, self-efficacy, and positive emotions (25, 35). Therefore, we assume that authoritative parents may entertain or actively interact with their kids in several ways, which could promote various motor skills. In contrast, grandparents may not vigorously interact with grandchildren during playtime (due to aging or physical fitness issues), which could result lower motor competence in children. Previous studies have shown that grandparenting had a negative effects on children's emotions, academic performance and other social behaviors (63, 64). A recent study from China reported that children grown-up in grandparents-headed families had lower creativity than those from families living without grandparents (42). This study further explained that the resources provided by parents are quite different from the resources provided by grandparents, which can influence creativity in children (42). Children with lower creativity or poor academic performance tend to be physically inactive, and displayed lower gross motor skills (65). Besides, childhood obesity among children aged 8–10 year in China is said to be associated with grandparenting caregiving style (66). Although the bodyweight of Chinese children (4–6 years) in our study was not significantly altered with caregiving style, other studies reported an association between obesity and poor motor skills in children. A systematic review identified an inverse association between motor competence and bodyweight status of children and adolescents (67). Increased fat mass is not only detrimental to motor competence performance (68) but also impede movement patterns of object projection skills that inherently demand high segmental velocities (69). Our findings further explained that authoritative parents may have some innovative approaches to help children to grasp new motor skills. Nevertheless, the influence of other parenting types, like authoritative, permissive, and uninvolved on motor skills of children (4–6 years) is yet to be investigated.

Due to the rapid migration of young parents from rural to urban areas in China (for better career or business opportunities), taking care of their child is a major concern in many families (23, 70). As a result, primary care of preschool children by grandparents is increasing in China (21, 23, 24). Grandparenting is associated with poor academic performance, poor creativity (63, 64) and lower physical activity among grandchildren (65). Less physical exercise and outdoor activities among children subsequently decrease physical fitness, weakened immunity, and even cause obesity (10). Previous studies have shown a strong positive correlation between children's FMS and physical activity (71–73). On the other hand, there is an erroneous perception among grandparents or elder community that academic activities are more important than physical activities or outdoor programs for children. It is therefore essential to understand the preschool children's motor competence, especially, who are taking care by grandparents. Furthermore, it is also imperative to study the motor skills of children from rural areas, where high percentage of grandparenting can be seen. Parenting without providing autonomous support may also lead to poor motor competence in children. Therefore, young parents particularly in uninvolved type, should prioritize their child's needs and feelings, and monitor their emotional and sedentary behaviors. Importantly, parents should avoid excessive screen time or social media usage, and need to engage with their child and praise their accomplishments. Additional activities like, taking children to parks, providing construction toys, weekend outdoor programs, and active co-participation in physical activities by caregivers could promote children's self-esteem and motor skills. Taken together, our findings emphasize that families and kindergarten schools need to pay attention and work collectively to provide children with more opportunities for physical activities.

### 4.4 Limitations

Preschool children participated in this study are Chinese, and our findings can be generalized to children in the Chinese society, where most caregivers are biological parents. Further studies are necessary to demonstrate the influence of similar caregiving styles on FMS of children from Western countries. The differences between biological parenting and non-biological parenting on motor skills can be studied in future to emphasize the importance of biological parenting. Our findings primarily addressed the influence of caregiving styles on children's FMS, however, the influence of other variables, including parental sex and household income on motor competence of children needs to be investigated. Furthermore, parenting type in this study was authoritative, and the influence of other parenting types, such as authoritative, permissive, and uninvolved on children's FMS is also need to be demonstrated. Finally, children in this study were from one caregiving style (parents or grandparents), and the effect of combination of two caregiving styles (both parents and grandparents) on children's FMS is also yet to be elucidated.

### 4.5 Practical implications

Poor motor competence observed in girl preschoolers with grandparenting implies that some practical interventions are necessary to promote their motor skills. Based on our findings, it is encouraged to practice positive parenting, particularly authoritative type to build a strong bonding between child and parent and to promote FMS proficiency. Furthermore, young parents should strive to balance their career and family, ensuring that they offer adequate care, facilities and support for their children.

## 5 Conclusion

Parenting caregiving style is strongly associated with proper development of FMS competence among kindergarten children. Improved FMS proficiency in children helps to enhance cognitive skills, academic performance, health-related outcomes and physical fitness. Therefore, parenting is highly encouraged for preschoolers, especially for girls, who reported poor motor skills with grandparenting caregiving style.

## Data availability statement

The original contributions presented in the study are included in the article/supplementary material, further inquiries can be directed to the corresponding authors.

## Ethics statement

The studies involving humans were approved by Institutional Ethical Committee of Zhejiang Normal University (ZSDR2019013). The studies were conducted in accordance with the local legislation and institutional requirements. Written informed consent for participation in this study was provided by the participants' legal guardians/next of kin.

## Author contributions

JH, SZ, and WY: data collection. JH and SZ: data analysis and original draft preparation. YZ, HZ, and LL: data interpretation and validation. WY, MK, and QC: supervision, project administration, review, and editing and finalize the manuscript. WY: funding acquisition. All authors have read and approved the final version of the manuscript.
